# Evaluating meaningful changes in physical functioning and cognitive declines in metachromatic leukodystrophy: a caregiver interview study

**DOI:** 10.1186/s41687-023-00595-7

**Published:** 2023-07-17

**Authors:** Susan Martin, Nimanee Harris, Dorothy Romanus

**Affiliations:** 1 RTI Health Solutions, Ann Arbor, MI USA; 2 Takeda Development Center Americas, Inc., Lexington, MA USA

**Keywords:** Arylsulfatase A, Caregiver, Enzyme replacement therapy, Gross motor function, Intrathecal, Lysosomal storage disease, Metachromatic leukodystrophy, MLD, Qualitative research

## Abstract

**Background:**

Metachromatic leukodystrophy (MLD) is a rare lysosomal storage disease caused by deficient activity of arylsulfatase A (ASA). Treatment options for patients are limited; gene therapy based on haematopoietic stem cell transplantation is the only approved treatment for some subtypes of MLD. Any therapeutic benefit of treatments must be meaningful for patients and their families. We evaluated the clinical meaningfulness of slowing the decline in gross motor function as measured by the Gross Motor Function Classification in MLD (GMFC-MLD) from the caregiver perspective via semi-structured telephone interviews with caregivers of children with late-infantile MLD. We also evaluated the perceived significance of declines in communication abilities measured by the Expressive Language Function Classification in MLD (ELFC-MLD). This work could help to inform the endpoints of a phase 2 clinical trial (NCT03771898) assessing the efficacy of intrathecal recombinant human ASA in MLD.

**Results:**

Twelve caregivers were recruited, reporting on 12 children with MLD. Children had a mean age of 6.1 years; mean age at symptom onset was 17.6 months. Most children (10/12) progressed from walking without support (categories 0–1) to a loss of locomotion (categories 5–6) in ≤ 2 years. Caregivers felt that GMFC-MLD and ELFC-MLD accurately described motor and language declines in their children, respectively. Most caregivers (10/12) reported that the idea of delaying disease progression would be meaningful. Further, a slowing of motor function decline in GMFC-MLD, from category 1 to category 3 or from category 2 to category 4 over 2 years, was seen as meaningful by all caregivers asked; however, only 3/12 caregivers reported that delayed decline would be meaningful if baseline category was ≥ 3. Caregivers also reported that delaying expressive language decline at any level that did not indicate a complete loss of expressive language (indicated by categories 1–3) would be meaningful.

**Conclusions:**

Caregivers of children with MLD felt that a delayed decline in gross motor function, as assessed by the GMFC-MLD, would be meaningful, supporting the selection of primary and secondary endpoints for the phase 2 clinical trial. Communication abilities were another area of significance for consideration in future clinical trial design.

## Background

Metachromatic leukodystrophy (MLD) is a rare, autosomal recessive lysosomal storage disease (LSD) characterized by a deficiency in the enzyme arylsulfatase A (ASA) [1]. The incidence is estimated to be between 1 and 40,000 and 1 in 170,000 in different populations [2]. Clinical manifestations of MLD can vary considerably across patients [2]. Three forms of MLD have been defined, distinguished by age at symptom onset: late-infantile (LI; onset < 30 months), juvenile (onset at 2.5 –< 16 years), and adult (≥ 16 years) [3]. LI MLD is the most common and rapidly progressing form of MLD. It generally manifests as gait disturbances initially, followed by a steep decline in motor and cognitive function [4–7]. Death typically occurs within 5 years from symptom onset for patients with LI MLD [2].

Several treatment approaches are currently in development for MLD [8], including intrathecal enzyme replacement therapy (ERT) with recombinant human ASA (rhASA; SHP611, now TAK-611) [9]. Although haematopoietic stem cell transplantation (HSCT)-based gene therapy with OTL-200 (Libmeldy™, Orchard Therapeutics) has been approved in some regions, it is only prescribed for patients with pre-symptomatic or early symptomatic juvenile MLD, or for patients with pre-symptomatic LI MLD [10, 11]. Therefore, it remains important to investigate ERT further as a therapeutic approach for symptomatic LI MLD.

A fundamental goal when developing treatment for disease is that any potential benefit translates to meaningful improvements for patients and their families. Consequently, input from caregivers or patients is valuable in the design of clinical trials and selection of study endpoints. Caregivers of patients with MLD have given valuable perspectives into our understanding of the natural history of MLD [7] and are well placed to provide insight on the value that a potential delay in motor and cognitive declines would bring to patients and their families following treatment [12]. The importance of collecting patient experience data during the drug development process, including the caregiver experience, has been recognized across stakeholders such as regulatory agencies and clinical trial researchers [13–15]. These data can be particularly important in the context of rare diseases, given that novel study designs and endpoints may be required owing to the limited number of available patients [16].

We report findings from caregiver interviews that explored the significance that a delay in functional decline would represent for children with LI MLD and their families. The primary objective was to evaluate caregiver perspectives on the clinical meaningfulness of delayed motor function decline as assessed by the Gross Motor Function Classification in MLD (GMFC-MLD). A secondary objective was to evaluate perceptions of the decline in communication abilities in children with LI MLD from a caregiver perspective. This information would help to inform the open-label, phase 2 clinical trial (NCT03771898), assessing the efficacy and safety of 150 mg doses of intrathecal rhASA administered weekly to children with LI MLD over a primary follow-up period of 2 years (106 weeks) [17]. The primary endpoint of the clinical trial is time to loss of locomotion (as indicated by category ≥ 5) during a 2-year follow-up period in patients aged 18–48 months with a GMFC-MLD of category 1 or 2 at baseline.

## Methods

### Study design and inclusion criteria

Twelve caregivers from the USA (all parents of a child with MLD) were identified by the MLD Foundation (a patient advocacy group willing to assist with recruitment) and recruited by RTI Health Solutions for telephone interviews (Table 1). To be eligible for the study, caregivers had to be caring for, or to have previously cared for, a child with a diagnosis of LI MLD, be ≥ 18 years of age, be able to speak and read English, and be willing to participate in a 1-hour telephone interview. The child must have had onset of the signs of MLD before 30 months of age and must not have undergone HSCT or bone marrow transplantation, or participated in any previous clinical trial. If no longer living (n = 1), the child must have been deceased for ≤ 6 months. If the caregiver was caring for more than one child with MLD (n = 1), they were asked to focus on the child who was born first. This approach was used with the aim of limiting the interview to 1 hour to reduce caregiver burden. This applied to one caregiver who provided care for twins, both with a diagnosis of MLD. This study was reviewed and deemed exempt by RTI Institutional Review Board.

**Table 1:** Characteristics of caregivers included in this study and their children with MLD

Characteristic	Total (N = 12)
Caregivers, N = 12	
Sex, n (%)	
Women	10 (83)
Men	2 (17)
Age	
Mean (range), years	37.4 (30–51)
Race/ethnicity, n (%)	
White	10 (83)
White/Asian	1 (8)
White/Pacific Islander	1 (8)
Employment, n (%)	
Full-time	6 (50)
Part-time	2 (17)
Not employed	4 (33)
Education, n (%)	
High school	2 (17)
Some college	5 (42)
College degree	5 (42)
Geographical region in the USA, n (%)	
Northeast	2 (17)
Southeast	4 (33)
Midwest	2 (17)
West	4 (33)
Children with MLD, N = 12	
Sex, n (%)	
Boys	7 (58)
Girls	5 (42)
Age at time of interview^a^	
Mean (range), years	6.1 (3–11)
Age at symptom onset	
Mean (range), months	17.6 (6–24)
Age at diagnosis	
Mean (range), months	29.8 (22–48)
Race/ethnicity, n (%)	
White	10 (83)
White/Asian	1 (8)
White/Pacific Islander	1 (8)

*MLD*, metachromatic leukodystrophy

^a^One child with metachromatic leukodystrophy was deceased; age at time of death was used

Note: not all percentages add up to 100, owing to rounding.

### Interview process

At initial contact by the MLD Foundation, a brief description of what would be asked during interview, the topic of interest, and eligibility criteria were provided. If caregivers expressed interest, their details were passed to RTI Health Solutions for a further screening process in which the interviews were then described in greater detail. Caregivers were informed that the interviews would focus on the experiences of their child with MLD, and those caregivers who wanted to participate provided verbal, informed consent. Each telephone interview lasted approximately 1 hour and was conducted by two team members experienced in conducting semi-structured interviews and qualitative research (SM, NH; RTI Health Solutions); one served as the primary interviewer, while the other took notes and monitored the need for additional questions or probes. All interviews were audio recorded, and participants were able to stop the interview at any time. Transcripts were reviewed by the RTI Health Solutions project team to ensure accuracy through a standardized technical and editorial process.

Interviewers followed a semi-structured guide, developed by the research team, to address the project objectives and to ensure a consistent approach across all interviews. The interviews began with a section of open-ended questions regarding caregiver observations and experiences of caring for a child with LI MLD and their experience of the timeline of MLD symptom progression. Following this, caregivers discussed aspects of their child’s physical functioning, and (time permitting) their expressive language abilities. Particular focus was placed on the interpretation of the GMFC-MLD and the Expressive Language Function Classification in MLD (ELFC-MLD) as instruments in describing disease progression; how these measures related to their child’s current physical functioning and communication abilities; the general meaningfulness of slowing progression as described by these measures; and the meaningfulness of slowing the progression of decline (specifically, of decline as defined by no greater than a two-category increase in the GMFC-MLD). The slowing of progression by no greater than a two-category increase in the GMFC-MLD was planned as the primary endpoint for the phase 2 clinical trial at the time of the caregiver interviews and remains a secondary endpoint. The current primary endpoint is a delay in time to loss of locomotion, as indicated by progression to a category ≥ 5 on the GMFC-MLD from a baseline category of 1 or 2.

The GMFC-MLD is a clinician-rated classification system of motor decline observed in MLD [18]. It describes seven categories of motor decline, representing clinically relevant stages of deterioration from normal (category 0) to loss of all locomotion with (category 5) or without (category 6) head control. The classification system has demonstrated a high level of inter-rater reliability. The ELFC-MLD is a clinician-rated classification system of expressive language decline observed in MLD describing five categories of language decline, from no impairment in expressive language (category 0) to a complete loss of even the use of single meaningful words (category 4) [6, 19].

### Data analysis

Descriptive statistics were used to summarize results for patient and caregiver characteristics. Qualitative data analysis followed researcher neutrality and systematic process. Specifically, a deductive framework following the themes contained in the interview guide was used by the research team (SM, NH) for each research objective to ensure accurate reflection of the results. One researcher led the transcript analysis (NH), and a senior reviewer (SM) was frequently consulted to seek agreement and confirm accuracy and reliability of the findings. Data tables were based on the content of the transcripts to display the concepts described during each interview and to document participant results across all interviews. Quotes included in this article have had identifiable information removed to ensure patient privacy is protected; redacted details and information that has been added for context are indicated with square brackets.

## Results

### Patient and caregiver characteristics

Twelve caregivers (all parents) took part in the interviews (Table 1). The mean age of caregivers was 37.4 years, and most (83%) were women. Two-thirds of caregivers (67%) were in full-time or part-time employment. The mean age of the children with LI MLD was 6.1 years at the time of the interview, and seven were boys. The mean age at symptom onset was 17.6 months, and mean age at diagnosis was 29.8 months.

### Caregiver descriptions of MLD onset and progression

All caregivers reported that the initial signs and symptoms of LI MLD that they noticed in their child were physical manifestations, such as gait irregularities, inability to walk, turned feet, crossed eyes, hand tremors, and leg pain. Walking issues were the most frequently observed initial signs, reported by 8 of the 12 caregivers.

Caregivers were easily able to recall and describe the progression of the physical functioning limitations of their child. Many of these descriptions included aspects of functioning assessed by the GMFC-MLD. For example, one caregiver described the quick decline in walking and standing ability, and its associated burden for her child: *“He would cruise around, like, the coffee table. He would hold my hands and walk, you know, as long as he held onto my hands and he had that support, he could do that. But he just didn’t have the physical strength to support himself. […] At 17 months, he started no longer cruising around things; he would pull up, but his little legs just kind of go weak. And so, he would just kind of stand, it was just literally like watching a baby going reverse.” (Interview 6).*

### Interpretation and relevance of the GMFC-MLD

Caregivers were asked to read each category of the GMFC-MLD (Table 2) aloud and describe the category in their own words. Overall, the categories defined in the GMFC-MLD were correctly interpreted by caregivers (illustrated by quotes in Table 2).

**Table 2:** Description of GMFC-MLD category definitions, with illustrative quotes from caregivers

GMFC-MLD category and description	Illustrative quotes from caregiver interview transcripts
0: Walking without support with quality of performance normal for age	• *Yes, I’d say he was walking and normal for age up until 18 months (Interview 9)* • *Yeah, so, with the walking without support, she was not … she was not able to do 0. So, I would say 0, she couldn’t do (Interview 11)* • *She probably was at that level for 5 months (Interview 1)* • *Yes. She hit all of her milestones, walking all the way up to two and a half would be … was normal for her (Interview 3)*
1: Walking without support but with reduced quality of performance (e.g., instability when standing or walking)	• *Yes, the age for that would have been … that winter, she would have been 2 [years old]. That would have been during the third … the start of the second month and into about the fourth month (Interview 3)* • *Yeah, number 1 definitely had applied. I would say he was about a year and a half to two and a half. Two or two and a half (Interview 7)* • *Walking without support was reduced. I would say no, because she never really could do … besides like cruising, she couldn’t really do 1 either. Walking with support. So, I would say with support maybe, but that would have to be like with one of us holding her hands to kind of help her (Interview 11)*
2: Walking with support. Walking without support not possible (fewer than 5 steps)	• *Support, that’s with his walker, so he was doing that through [date removed]. Or not all the way through [date removed], but I know through [date removed], so 28 months. 28, 29 months (Interview 9)* • *She was able to for every so often. It would have been around [date removed], so I guess that would be two and a half years old, she was still able to, but she depended on us for help, and that pretty much lasted until … I would say until about [5 months later] (Interview 3)*
3: Sitting without support and locomotion such as crawling or rolling. Walking with or without support not possible	• *That I would say, she could really only sit without positioning with pillows and things like that from maybe 3 … the first 3 months of diagnosis, maybe like 3 months from diagnosis (Interview 5)* • *Sitting without support, he did that … he only did that through [date removed], so that was only 1 more month, so 30 months (Interview 9)*
4a: Sitting without support but no locomotion OR 4b: Sitting without support not possible, but locomotion such as crawling or rolling	• *[4a] Yeah. Yeah, I’d say that, probably did for a period of time. I’d say she probably did that for like maybe through May possibly June (Interview 1)* • *[4a] Yeah. And again, that would have been like right around the same time because that sort of progression was really rapid (Interview 11)* • *[4b] No, I would say that once the ability went, there was really no more, trying to move her body on the floor after that (Interview 11)* • *I would say 4b is relevant. Probably 6 months (Interview 1)* • *[4a] No. I don’t think so. I think she needed… still crawl or roll around when she could sit unsupported (Interview 8)*
5: No locomotion nor sitting without support, but head control is possible	• *That was definitely relevant. That was kind of the last thing she could move was her head. And I would say that brings her up to about 6. Maybe another 6 months or so, she could still kind of turn her head back and forth (Interview 1)* • *So that was pretty much, that was pretty much [date removed], yeah, that was by her [event removed] so 5 or 6 months out (Interview 5)* • *5 I think is where he’s at now, and then 6, not yet (Interview 7)*
6: Loss of any locomotion as well as loss of any head and trunk control	• *Head and trunk control were just the steady decline like she couldn’t … yes she had lost her control but she has lost more and more of her control. Like in the beginning, she used to kind of rock teeter-totter and rock to try to hold herself up, you know her head, now she might be able to turn her head slightly, definitely can’t hold it up (Interview 5)* • *I’d say it was probably by about [date removed], she really needed full support on her body. She was, by then, she had a neck brace to support … to help hold her head up (Interview 8)* • *He is a 100% dependent on somebody to move him, move his arms, move his head for positioning. He hasn’t smiled for me since [date removed]. His eyes are still … he twinkles with his eyes (Interview 9)*

*GMFC-MLD*, Gross Motor Function Classification in metachromatic leukodystrophy

All caregivers stated that the GMFC-MLD was an accurate description of their child’s physical functioning declines. One caregiver summarized: *“I think that in general kind of encompasses, from what I’ve seen from other families and heard, you know, there were some kids that walk and were normal. And there were some that didn’t. But I feel like […] all these descriptions probably happened to most people in one way or another. So yeah, they all make sense to me. I think they capture basically what happens.” (Interview 4).*

Most caregivers (n = 10) also reported that their child progressed from category 0 or 1 to a loss of most or all locomotion (categories 5 or 6) in ≤ 2 years, with the most rapid decline in function being reported as a period of 4–5 months. The remaining two caregivers reported progressions of longer than 2 years; one estimated 2.5 years for their child’s progression to category 6, and the other reported a period of approximately 3 years before progression to category 5. All caregivers stated that their child was at either category 5 or category 6 at the time of the interview (or at the time of death for the one child who was deceased): two at category 5, nine at category 6, and one at a category that the participant felt was between categories 5 and 6.

### Meaningful delay in progression of physical functioning declines

Caregivers were asked how they felt about specific hypothetical delays in motor function decline, as measured by the GMFC-MLD (Table 3). Ten of the 12 caregivers reported that the idea of delaying or slowing down their child’s disease progression would be meaningful. Caregivers felt this delay could allow their child more time to enjoy their lives without the progressive pain or confusion that accompanied their decline: *“Oh, yeah. Because then we can have them more comfortable longer. If it will start slowing down, they’re not going to be in constant pain all the time. They’ll have breaks where they can actually enjoy things.” (Interview 2).*

**Table 3:** Summary of caregiver perspectives on specific hypothetical delays in motor function declines

Finding	Caregivers, n (%)
Agreed that slowing overall physical disease progression is meaningful (N = 12)	10 (83)
Agreed that slowing of progression as defined by no more than a 2-category increase in GMFC-MLD after 2 years is meaningful, if baseline is category 1 or 2 (N = 11)	11 (100)
Agreed that slowing of progression as defined by no more than a 2-category increase in GMFC-MLD after 2 years is meaningful, if baseline GMFC-MLD category was 3 or greater (N = 12)	3 (25)
GMFC-MLD category considered most valuable to preserve for longer before further disease progression (N = 12)	
Unspecified 1 2 3 4 5 6	1 (8) 0 (0) 8 (67) 2 (17) 1 (8) 0 (0) 0 (0)

*GMFC-MLD*, Gross Motor Function Classification in metachromatic leukodystrophy

*“Absolutely. I would have loved more time with her even being able … even with her having the decrease in walking, I would [have] enjoyed every minute of it. Watching it happen the way it happened, and watching your child not understand why they’re not able to walk anymore, or they’re not able to do the things that they were able … once able to do, whether it be a week ago or a month ago, in her terms, it’s devastating. And it’s one of those things that I would never want to have to watch again, but at the same time, giving her time to at least slow the progression and her at least enjoy a little bit more of her time of doing it, that would mean the world to me and I’m sure it means the world to her.” (Interview 3).*

Of the two caregivers who did not feel that delaying or slowing down their child’s physical disease progression would be meaningful, one felt that a delay in disease progression could prolong their child’s suffering, and the other felt that a delay in physical progression would be less important if not accompanied with a slowing of their cognitive declines: *“Yeah, of course, it would be nice you know if he could still sit up and hold his head up and all the things. But it’s hard to say that because none of that matters if the mental part could be saved or put off or whatever.” (Interview 4).*

Most caregivers (n = 8) reported that GMFC-MLD category 2 was the most desirable category (excluding category 0) within which to gain additional time before further progression. This was because this category was often associated with a time when their child was still happy and able to engage in play: *“Oh, 2 for sure. Because when they walked they were happy.”*

*“I would say 2. Because when he was […] in stage 2, walking with support, he was still normal mentally. And we still played and all that stuff.” (Interview 4)*. One of these caregivers reported that they had selected category 2 over category 1 for safety reasons: “*Almost with him walking with the walker, he’s a little safer than struggling to walk and falling all the time.” (Interview 9).*

Two caregivers selected category 3 and one selected category 4a, but for similar reasons; these reflected levels at which the child was still able to enjoy life: *“So at any point up until she totally couldn’t move or enjoy any part of life. We would’ve stopped it. We would’ve taken more time for sure, if we had it.” (Interview 1).*

One caregiver did not specify a category but simply stated, *“anywhere where she is not suffering.” (Interview 1).*

### Meaningfulness of GMFC-MLD increase of no more than two categories

Caregivers were next asked about decline defined as no greater than a two-category increase from baseline in GMFC-MLD over 2 years; this definition is consistent with one of the secondary endpoints of the phase 2 clinical trial of intrathecal rhASA (in which the baseline is required to be category 1 or 2) [17]. Eleven of the 12 caregivers were asked if a slowed progression, as represented by going from either category 1 to 3 or from category 2 to 4 over a period of 2 years, would be meaningful. The remaining participant was only asked about slowing progression beginning at category 3 or 4.

All 11 caregivers who were asked agreed that this extent of slowing in disease progression would be meaningful to them. A common theme for why caregivers felt that this delay would be meaningful related to patients and caregivers having more time in a *“healthier phase”*, in which the children with MLD were happy and were experiencing less pain: *“I think, compared to where it’s at now, yes [category 2 to category 4]. Because […] with my child I think she could still experience kid stuff and don’t worry, be happy and play.” (Interview 8).*

*“I think so, yes. And I think along with that I feel […] if we could stop at that, or slow down at that level [category 3], I feel like then she wouldn’t be doing this extreme hypotonia and all of the pain associated with that. And I think that would be very, very meaningful.” (Interview 10).*

One caregiver also expressed a feeling that a delay in disease progression at this stage might allow time for additional treatment options to become available that could help her child: *“That would be amazing [categories 1–3]. Well, because those first 6 months, even though there was such a landslide, they were like trying to get everything we could before it was gone and can have it longer would be I don’t know what the words would be but it would also give […] time, time together, time to live, time maybe more treatment or cures or something.” (Interview 1).*

Three of these 11 caregivers reported that, although the suggested slowing of physical decline would be meaningful, it was just as important or more important to consider the child’s other abilities, such as cognition and communication. One caregiver further noted the importance of additional physical issues that accompanied progression beyond mobility: *“Yeah. … So if slowing down the physical progression to the example you said were to happen [from category 1 to category 3], I would imagine the mental progression would also try to time with that. So … I know we’re not talking about that [cognitive decline], but that’s all I can really think about because again, I don’t care if he’s physically disabled. […] Yes, it would be meaningful [slowing of physical decline]. But again, I go back to just having [name deleted] be able to talk to us, and play with us, and stuff like that.” (Interview 4).*

When asked about whether a similar extent of delayed progression would be meaningful if their child had more advanced physical dysfunction to start with, represented by GMFC-MLD category 3 or greater, one caregiver felt that any extra time with their child, wherever that may fall, would mean a lot: *“[From category 3 to category 5] I would say that any type of way where you can kind of slow things or preserve things … 2 years … I mean that’s … with her, everything was gone before then. So yeah, I think anyway, anywhere you fall, if it’s possible to slow the progression, then yes.” (Interview 11).* However, only two others reported feeling similar. The remaining nine caregivers were either uncertain or did not believe this would be a meaningful change. The impact of delayed progression at a more advanced stage of MLD on quality of life was a common theme in responses: *“Okay. Well, I feel like that’s a yes and no. That’s hard. That kind of puts me on the fence, the kind of scary … scary one to answer. I guess it’s, only … then you have to think about, is it 6 months of suffering or a little bit of halting? That’s a hard one.” (Interview 10).*

*“[From category 3 to category 5] Yeah, once he hit the 5, I don’t think it’s important to slow it. […] It’s again, quality of life, for his enjoyment. […] When he was at the 5, his body seems to be … he’s really, really rigid, he’s very stiff. He’s mostly comfortable in his wheelchair sitting up. He’s uncomfortable, he’s medicated to try to relax his muscles, and medicated for nursing and all these other things. So, don’t want to kind of extend life just to keep him drugged up all the time so he’s not in all kinds of pain.” (Interview 7).*

*“[From category 3 to category 5] I wouldn’t see that being quite as successful because at a 5, there’s still no real quality of life. So I guess I probably wouldn’t necessarily see that being successful.” (Interview 1).*

The feedback that slowing functional decline is less meaningful once a child reaches GMFC-MLD category 5 generally supports the primary endpoint of a delayed time to loss of locomotion.

### Interpretation and relevance of the ELFC-MLD

Nine of the 12 caregivers were asked for input on the ELFC-MLD; the remaining three were not asked owing to time constraints. As for the GMFC-MLD, caregivers were asked to read aloud and describe the categories of the ELFC-MLD (Table 4) in their own words.

**Table 4:** Description of ELFC-MLD category definitions, with illustrative quotes from caregivers

ELFC-MLD category and description	Illustrative quotes from interview transcripts
0: Communicates in complete sentences at a quality and performance normal for age	• *This was relevant all the way up till the age of 2 years old and 7 months. I would say at 2 years old and 7 months is when we started noticing the decline so from before then, everything she could complete in full sentences, talk to you, didn’t know a stranger, talked to everybody (Interview 3)* • *That would apply to her, yeah (Interview 5)* • *Yes. Probably really from the time she started. Let’s say 2½ years, 2 years (Interview 1)*
1: Communicates in complete sentences at a reduced quality and performance for age	• *Yes, and that would probably be 26 to 28 months. So, for about 2 months, I’d say it was kind of declining (Interview 4)* • *Yes and I’d say she did that, it’s hard to say when her sentences kind of weaned off and I’d say may be for few months maybe 1 month. 1 to 2 months (Interview 5)*
2: Cannot communicate in complete sentences, but able to use 2-word phrases	• *Yes but only for, really only for a couple months. Cause he kind of went … he went from saying more to just saying those one words (Interview 6)* • *Yeah. I’d say that was probably around [date removed]. So, she would have been 27 months, 28 months (Interview 8)*
3: Cannot communicate in 2-word phrases, but able to use single, meaningful words/ideas	• *Maybe just like the yes/no kind of stuff. [Date removed] he was … a little bit after 4. And 4 was, yeah, I would say like [date removed], around there (Interview 7)* • *Sorry, I’m flashing back in my memory. Yeah, I think he did. Like I think … I think he did for a very short time (Interview 4)*
4: Complete loss of expressive language	• *So, complete loss of expressive language, which … that happened so rapidly. She’s been like that for years (Interview 11)* • *And then she’s been ‘complete loss of expressive language’, gosh for probably 5 years now (Interview 1)*

*ELFC-MLD*, Expressive Language Function Classification in metachromatic leukodystrophy

All nine caregivers were able to interpret each category of the ELFC-MLD and stated that the system provided an accurate description of their child’s expressive language declines (indicated by quotes in Table 4). All stated that their child’s current expressive language level was at category 4, a complete loss of expressive language.

### Meaningful delay in progression of expressive language declines

All nine caregivers generally felt that gaining additional time at any level that did not indicate a complete loss of expressive language (categories 1–3) would be meaningful. One caregiver felt that a reason for this was the importance of the child being able to communicate their needs to other people: *“Yeah, I mean, all the way to 3 for sure. Because, […] they can still interact. I think when they can’t communicate, they get very frustrated. They’re different. I feel like I can tell what he wants pretty good, because I’m his mom. But if he’s at school, the nurses or other people, […] he would really struggle to get things across. So, I know them being … people not being able to communicate what they need and stuff is very frustrating for that person.” (Interview 6).* Seven caregivers were asked about delaying the decline in progression from a category 1 to a category 2 or 3 over 2 years, and all seven reported that this would be meaningful to them. For some caregivers, the importance of being able to say even just one word meant that their child would be better understood: *“Yeah, I would say so, definitely. Since you can verbally be able to answer probably yes or no or call for parents, say hi. If it’s just simple things like that, that’s huge.” (Interview 11).*

*“Yeah, yeah. Because again, he would still be able to hopefully get across a little bit of what he’s feeling. Whether it’s hot, or hungry, or pain, or whatever. Just to kind of still tell us what he needs and how we can help him.” (Interview 4).*

Another caregiver described the overall significance of a delay in the decline of expressive language related to just being able to hear their child’s voice: *“100% yes. Not to cut you off but 100% yes, to hear her voice. I would give anything to be able to hear her voice for 2 more years. That was the most precious thing that I would say that I ever lost. Even with her losing her mobility, … her voice was the most precious and being able to hear her call my name or anything, I absolutely would give anything to slow that progression.” (Interview 3).*

## Discussion

Overall, caregivers found that the GMFC-MLD and ELFC-MLD were easy to understand and reflected their experiences of their child’s physical functioning and language losses associated with disease progression in LI MLD. Caregivers endorsed the general importance of prolonging the time to motor function decline and felt that no more than a two-category increase in GMFC-MLD from baseline over 2 years would represent a meaningful slowing in the progression of motor function losses caused by LI MLD. This supports the selection of both the primary and secondary endpoints of the phase 2 trial of intrathecal rhASA and aligns with previous findings on the relevance of motor function for quality of life in people with LSDs [12, 20]. Participants also indicated that the baseline level of GMFC-MLD at the time of treatment was a key factor in determining the meaningfulness of a delay in progression, with only three caregivers reporting that a delay in an increase of no more than two categories over a period of 2 years (representing a worsening of function) would be meaningful if the child started treatment when at category 3 or greater.

All caregivers described observing a sharp deterioration in motor function in their child from first manifestations of gait disturbances and balance issues through to a complete loss of any head or trunk control. These reports agree with our current knowledge of the clinical progression of LI MLD [5–7], which tends to show a more homogeneous progression across patients than other subtypes [21] and supports the notion that these results are generalizable to patients with LI MLD. The results also support previous literature that suggests that physical symptoms such as immobility and respiratory issues are considered the most burdensome [12]. Caregivers noted a similar burden, describing the continuous increase in need for mobility support for their child, as well as details of increasing respiratory complications as the disease progressed. Our findings also highlighted the wide-ranging consequences that result from motor decline in these patients; caregivers noted the distress that such motor dysfunction caused for their child, including confusion and anxiety, and emphasized the emotional difficulties of seeing their child in increasing pain and distress. This work adds a deeper understanding of the personal significance that a delay in such declines in motor function represents for patients and their families; it also supports the relevance and accuracy of the GMFC-MLD as a measurement tool for clinical trials.

An important point that emerged from the interviews was that some caregivers feel that it is just as important, or more important, to slow other forms of disease progression, such as communication decline, cognitive decline, or physical issues that cause discomfort (e.g., spasticity). For instance, a number of caregivers noted that the decrease and eventual loss of the ability to swallow or eat independently was particularly burdensome. This is not captured in the GMFC-MLD.

Cognitive decline is known to be a significant accompanying symptom to motor deficits in LI and other forms of MLD [22]. Two caregivers said that a slowing of motor decline may not be meaningful if it were not accompanied by a slowing of cognitive decline. This study provides an indication that maintenance of communication remains an additional priority for caregivers, as evidenced by most caregivers agreeing that it would be meaningful to gain time within any category of the ELFC-MLD that did not represent a complete loss of expressive language. It is important to note that variation in ELFC-MLD score is likely to be limited in this cohort given their young age, and we were unable to interview all caregivers on this aspect owing to time constraints. Nevertheless, the overarching agreement on the meaningfulness of this facet highlights the significance of delaying communication decline and the importance of considering language function when assessing efficacy of new therapies in development.

Overall, there was support for the key phase 2 clinical trial endpoints to assess efficacy of treatment with intrathecal rhASA. Support from patient and caregiver perspectives is particularly important in rare diseases, in which small study numbers can limit our understanding of quantitative endpoints [16]. The findings presented here further highlight the importance of considering baseline function when defining the trial endpoint; the impact on quality of life of delaying further decline at an already advanced stage of disease was often a concern for caregivers. Terminal stages of disease have been documented as the most distressing periods for caregivers [23], so delaying progression once the patient has reached these advanced stages may place an additional burden on the family unit. Furthermore, in line with indications that early intervention is likely to improve therapeutic effectiveness in LI MLD [19, 24, 25], early intervention is also seen as meaningful from the caregiver perspective in terms of maintaining a level of functioning relevant for improved quality of life for patients and their families.

Although this study allowed us to obtain detailed feedback from a caregiver population, it is important to consider potential limitations. Owing to the nature of rare diseases, the sample size for this study was small, all caregivers were recruited from the USA, and the majority of caregivers were white. This might limit the generalizability of these findings to the global population of patients with LI MLD. In addition, the interview process relied on the retrospective recollections of caregivers for disease progression timings, which may be susceptible to recall bias. Therefore, the timings reported here should be considered as approximations. Finally, there was some variability in interview questions covered for each caregiver. This was mainly because of the open-ended nature of the interviews and, in some cases, time restrictions. Despite these limitations, the findings are in line with prior literature and provide valuable insights into caregiver perspectives of disease progression in MLD.

## Conclusions

These results suggest that the concept of delaying the declines in physical functioning experienced by children with LI MLD is meaningful for caregivers and they highlight the value of obtaining caregiver input throughout the development of clinical trials in rare diseases. Specifically, caregivers found the GMFC-MLD to be interpretable and relevant in describing the declines in physical functioning experienced by their children, and their feedback suggests that the endpoints included in the phase 2 clinical trial of intrathecal rhASA (NCT03771898) using this tool would be a meaningful measure of efficacy.

## Data Availability

De-identified individual participant data from this particular study will not be shared as there is a reasonable likelihood that individual patients could be re-identified (due to the limited number of study participants).

## Abbreviations

ASA: Arylsulfatase A
ELFC-MLD: Expressive Language Function Classification in metachromatic leukodystrophy
ERT: Enzyme replacement therapy
GMFC-MLD: Gross Motor Function Classification in metachromatic leukodystrophy
HSCT: Haematopoietic stem cell transplantation
LI: late-infantile
LSD: Lysosomal storage disease
MLD: Metachromatic leukodystrophy
rhASA: Recombinant human arylsulfatase A.
